# Night-eating syndrome is associated with food consumption frequency among Chinese college students

**DOI:** 10.1038/s41598-026-36505-2

**Published:** 2026-01-17

**Authors:** Zixuan Hao, Xiaoqin Guo, Qi Jing, Boyang Zhao, Mengyi Huang, Zhongyu Ren

**Affiliations:** 1 School of Physical Education , Southwest University , Chongqing, China; 2 Sports Science Research , Sendai University , Sendai, Japan

**Keywords:** Food consumption patterns, Night eating syndrome, Chinese college students, Cross-sectional study, Epidemiology, Risk factors

## Abstract

Patients with night-eating syndrome (NES) exhibit circadian rhythm dysfunction and comorbidities, including insomnia, anxiety, and depression. Therefore, understanding its potential risks and consequences is crucial. This study examined associations between night-eating syndrome (NES), food consumption frequency, and the frequency of consuming three daily meals among Chinese college students. A total of 11,856 university students (mean age 18.8 years) participated in this large-scale cross-sectional study. NES was assessed using the Nocturnal Eating Questionnaire and categorized as no NES (score < 25), mild NES (25–30), or severe NES (> 30). Food consumption frequency was evaluated across five categories—fruits, vegetables, fast food, snacks, and sugary beverages. The frequency of consuming breakfast, lunch, and dinner was also recorded. Multivariate logistic regression analyses were used to examine associations between NES severity and food consumption frequencies and meal-consumption frequency. After adjusting for confounders, NES was positively associated with higher consumption of fruits, vegetables, snacks, and sugary beverages, and with more frequent breakfast and lunch consumption. However, no significant associations were observed for fast food or dinner frequency. These findings indicate that NES is associated with distinctive eating patterns among college students and underscore the importance of considering NES when evaluating dietary behaviors in this population.

## Introduction

In 1955, Stunkard first reported that patients with night-eating syndrome (NES) exhibited circadian rhythm dysfunction^1^, suggesting an imbalance between eating and sleep patterns^2^. The syndrome is defined as a daily intake of > 25% of the recommended calorie intake after dinner, accompanied by nocturnal eating at least twice a week for a minimum of 3 months^3^. It is characterized by morning anorexia and hyperphagia in the evening (awakening to eat during the night), along with difficulties in both falling asleep and maintaining sleep^1^. NES is associated not only with physical conditions such as obesity and diabetes^4,5^, but also with a range of other health issues, including insomnia, depression, and anxiety^6^. These comorbidities can significantly affect overall well-being and quality of life. Thus, examining the association between NES and lifestyle, particularly dietary habits, is crucial for understanding its potential risks and consequences.

Individuals with NES often display irregular nighttime eating habits, which may influence daytime eating behaviors and contribute to an overall imbalanced diet.Previous studies in patient populations and healthy adults have demonstrated significant correlations between NES and specific dietary preferences. For example, a study of obese individuals in Pakistan revealed a positive correlation between NES and frequency of daily snacking^7^. Another study in adults revealed that NES was positively associated with unhealthy dietary habits, such as the consumption of sugary beverages and fast food, and negatively associated with healthy eating habits, such as the consumption of fruits and vegetables^8^. Furthermore, the association between NES and breakfast frequency has been explored in studies involving Bangladeshi university students, Turkish pregnant women, and patients with type 2 diabetes in the United States. These studies reported a statistically significant inverse association between NES and breakfast intake^5,9,10^, while no significant associations were observed for lunch or dinner consumption in patients with type 2 diabetes^5^.

A significant proportion of university students engage in unhealthy eating patterns at night. Previous research indicates that 57.10% of university students regularly consume late-night snacks, with 6.79% eating late-night snacks every night, 23.15% eating late-night snacks frequently, and 27.16% eating late-night snacks occasionally^11^. The university years represent a critical period in the transition from adolescence to adulthood^12^ and for the development of long-term eating habits, which can influence both short-term and chronic health outcomes. Despite several studies investigating the association between NES and dietary behaviors, there remains a lack of research examining associations between NES, food consumption frequency, and meal regularity among Chinese populations, particularly among college students. Accordingly, this study used a cross-sectional design to examine the association between NES and both single and multiple dietary consumption frequencies. Moreover, this study aims to provide data that may inform strategies for promoting healthier dietary habits and identifying target populations for potential interventions among Chinese college students.

## Methods

### Participants

This study employed an ongoing cohort design to identify protective and risk factors for physical fitness among Chinese university students. The survey locations were selected based on the physical fitness testing centers of 11 universities in China, including six in the province of Liaoning, one in the city of Jilin, and four in the municipality of Chongqing. The selected universities offer a diverse academic portfolio comprising disciplines such as philosophy, logic, economics, finance, business and trade, political science, sociology, early childhood education, elementary education, Chinese language and literature, and foreign languages and literature. A combination of convenience and cluster sampling was employed to select 1–2 grades. A total of 11,856 university students participated.

The study was approved by the Ethics Committee of the School of Physical Education, Southwest University, and conducted in accordance with local regulations and institutional requirements. Participants provided written informed consent after being fully informed of the study purpose, significance, and precautions; parental consent was obtained for participants aged < 16 years.

### Assessment of NES

The validated Chinese version of the Night Eating Questionnaire (NEQ) was used to assess NES severity^13^. The NEQ consists of 14 items, each scored from 0 to 4. Scores for all items except question 13 were summed, resulting in a total score ranging from 0 to 52, with higher scores indicating greater NES severity. Based on a previous study^13^, an NEQ score of < 25 was classified as normal, ≥ 25 but < 30 as mild NES, and > 30 as severe NES. The internal consistency reliability of the Chinese version of NEQ was validated by calculating the Cronbach’s alpha coefficient, which yielded a value of 0.711. This result indicates that the Chinese version of NEQ exhibits a satisfactory level of internal consistency.

### Assessment of food consumption frequency

Participants were asked to indicate their consumption frequency for five food categories: fruits, vegetables, fast food (e.g., pizza, burgers), snacks (e.g., chips, crisps), and sugary beverages (e.g., soft drinks and sugary fruit drinks)^14^. For each food type, respondents indicated their level of consumption frequency on a five-point Likert scale, where 1 = strongly dislike, 2 = dislike, 3 = neutral, 4 = like, and 5 = strongly like^15^.

For healthy dietary items (fruits and vegetables), participants who selected “strongly dislike,” “dislike,” or “neutral” were assigned 1 point, while those who selected “like” or “strongly like” received 0 points. In contrast, for unhealthy dietary items (fast food, snacks, and beverages), participants who chose “like” or “strongly like” were assigned 1 point, while those who selected “strongly dislike,” “dislike,” or “neutral” were assigned 0 points.

Additionally, respondents were asked to indicate the frequency of consuming three meals a day (breakfast, lunch, and dinner) on an eight-point scale: never, one time per week, two times per week, three times per week, four times per week, five times per week, six times per week, and daily^16^. For analysis, participants consuming meals for ≤ 6 days per week were considered irregular (coded 1), and those consuming meals 7 days per week were considered regular (coded 0). Table 1 shows the questions and options regarding dietary consumption frequency and the frequency of three daily meals.

**Table 1:** Description of dietary consumption frequency questionnaire.

Item 1: Do you like to eat fruits?							
strongly dislike	dislike	neutral	like	strongly like			
Item 2: Do you like to eat vegetables?							
strongly dislike	dislike	neutral	like	strongly like			
Item 3: Do you like to eat fast food (e.g., pizza, hamburgers)?							
strongly dislike	dislike	neutral	like	strongly like			
Item 4: Do you like to eat salty snack food (e.g., potato chips)?							
strongly dislike	dislike	neutral	like	strongly like			
Item 5: Do you like to eat soft drinks and sugared fruit drinks?							
strongly dislike	dislike	neutral	like	strongly like			
Item 6: How many times a week do you have breakfast?							
never	1	2	3	4	5	6	7
Item 7: How many times a week do you have lunch?							
never	1	2	3	4	5	6	7
Item 8: How many times a week do you have dinner?							
never	1	2	3	4	5	6	7

### Assessment of covariates

All participants completed a self-designed questionnaire that assessed several demographic variables, including sex, age, annual household income (divided into three categories: < RMB20,000, RMB20,000–RMB35,000, and > RMB35,000), place of residence (urban or rural), and parental education level (classified into no formal education, completed primary school, completed junior high school, completed high school, or completed college or above).

### Statistical analysis

All data were imported into an Excel database and analyzed using SPSS version 23.0. Continuous variables are presented as means ± SD, and categorical variables as percentages (%).

Multivariate logistic regression models were constructed using NES categories (normal group as the reference) as the exposure variables and each single dietary consumption frequency as the outcome variable. Confounding factors were added to the models separately to obtain odds ratios (ORs) and 95% confidence interval (CIs). Model 1 was a crude model, Model 2 was adjusted for sex, age, annual household income, place of residence, and parental education levels, and Model 3 was further adjusted for sleep duration, sleep quality, and depression index based on Model 2.

Subgroup analyses were conducted to examine the association between NES and different dietary consumption frequencies and to explore the consistency of results among different subgroups. All statistical analyses were performed using two-sided tests, with *P* < 0.05 indicating statistical significance.

## Results

Table 2 presents the sociodemographic characteristics of the participants. A total of 11,856 college students participated, of whom 33.3% were male, with a mean age of 18.8 years. Among participants, 34.6% had an annual household income of > 35,000 yuan, and 58.9% resided in rural areas. Approximately one-third had parents with a bachelor’s degree or higher.

**Table 2:** Demographic characteristics of participants according to the level of night eating syndrome (NES).

	Night eating syndrome groups				*P* value
	Total (11,856)	Normal (10,883)	Mild (816)	Severe (157)	
Age (Mean ± SD)	18.8 ± 1.2	18.8 ± 1.1	19.2 ± 1.3	19.0 ± 1.2	< 0.001
Sex, %					< 0.001
Male	33.3	32.4	42.2	42.2	
Female	66.8	67.6	57.8	59.9	
Annual family income (RMB), %					0.002
<20,000	35.5	35.2	38.1	48.4	
20,000–35,000	29.9	30.1	27.1	27.4	
>35,000	34.6	34.7	34.8	24.2	
Place of residence, %					0.195
Town	41.1	40.9	44.1	41.4	
Rural area	58.9	59.1	55.9	58.6	
Father’s education, %					0.537
Primary and below	23.9	24.1	21.7	20.4	
Junior high	39.5	39.4	40.6	42.7	
High school	21.4	21.4	21.7	19.1	
Junior college and above	15.1	15.0	15.0	17.8	
Mother’s education, %					0.147
Primary and below	31.9	32.0	31.5	28.7	
Junior high	36.4	36.6	34.8	31.2	
High school	18.6	18.3	21.1	24.8	
Junior college and above	13.1	13.1	12.6	15.3	

Continuous variables are presented as means ± SD, while categorical variables are expressed as percentages. P-values were calculated using analysis of variance for continuous variables and chi-square tests for categorical variables

**Fig. 1:**
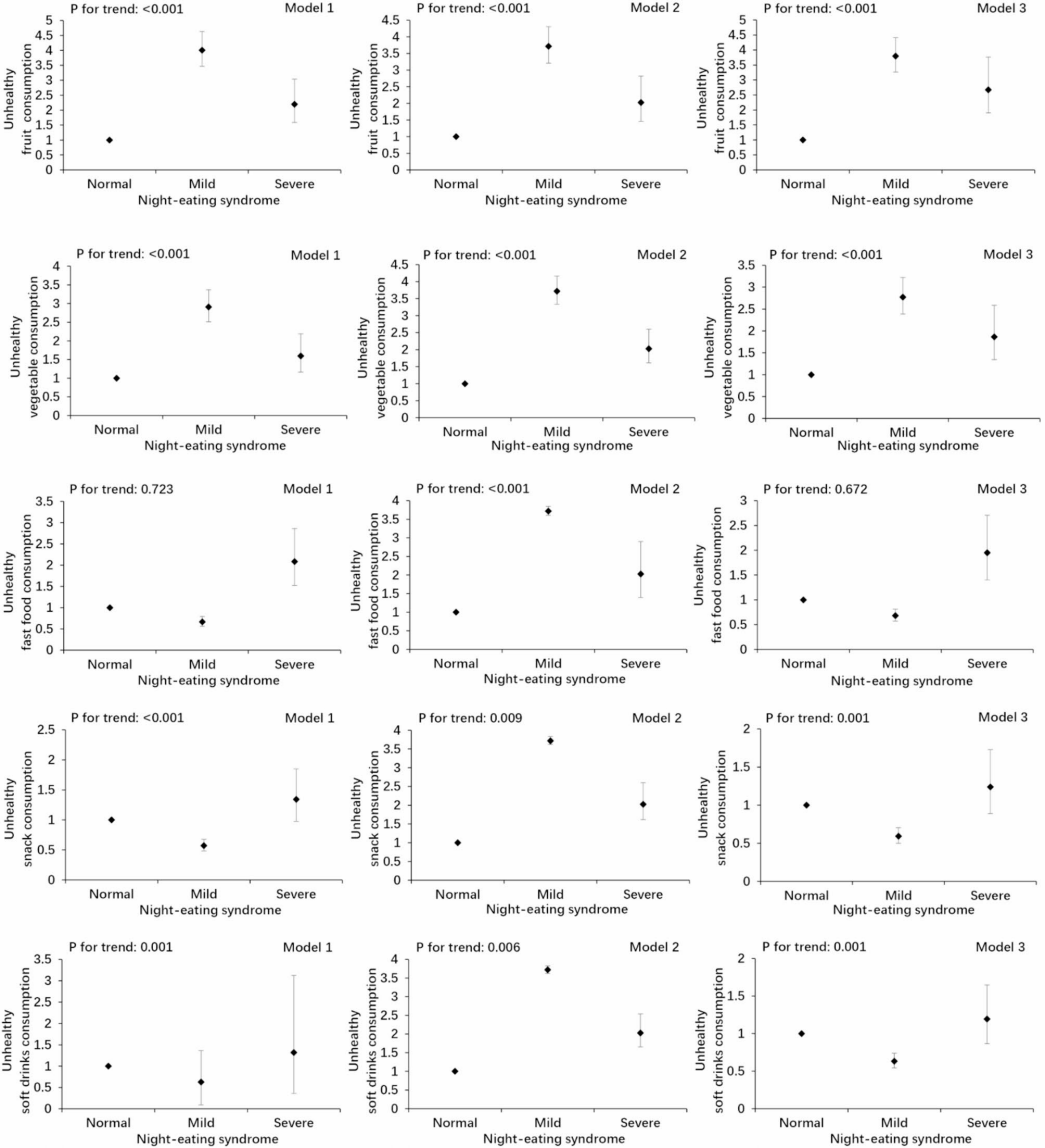
Multivariate logistic regression of food consumption frequency according to night eating syndrome. Model 1: crude; Model 2: adjusted for sex, age, annual family income (< 20000, 20000–35000, > 35000 yuan), place of residence (town or rural), and parental education (primary and below, junior high, high school, or junior college and above); Model 3: adjusted for Model 2 variables plus sleep duration, sleep quality, and depression index. Adjusted data are expressed as odds ratio (95% confidence intervals).

**Fig. 2:**
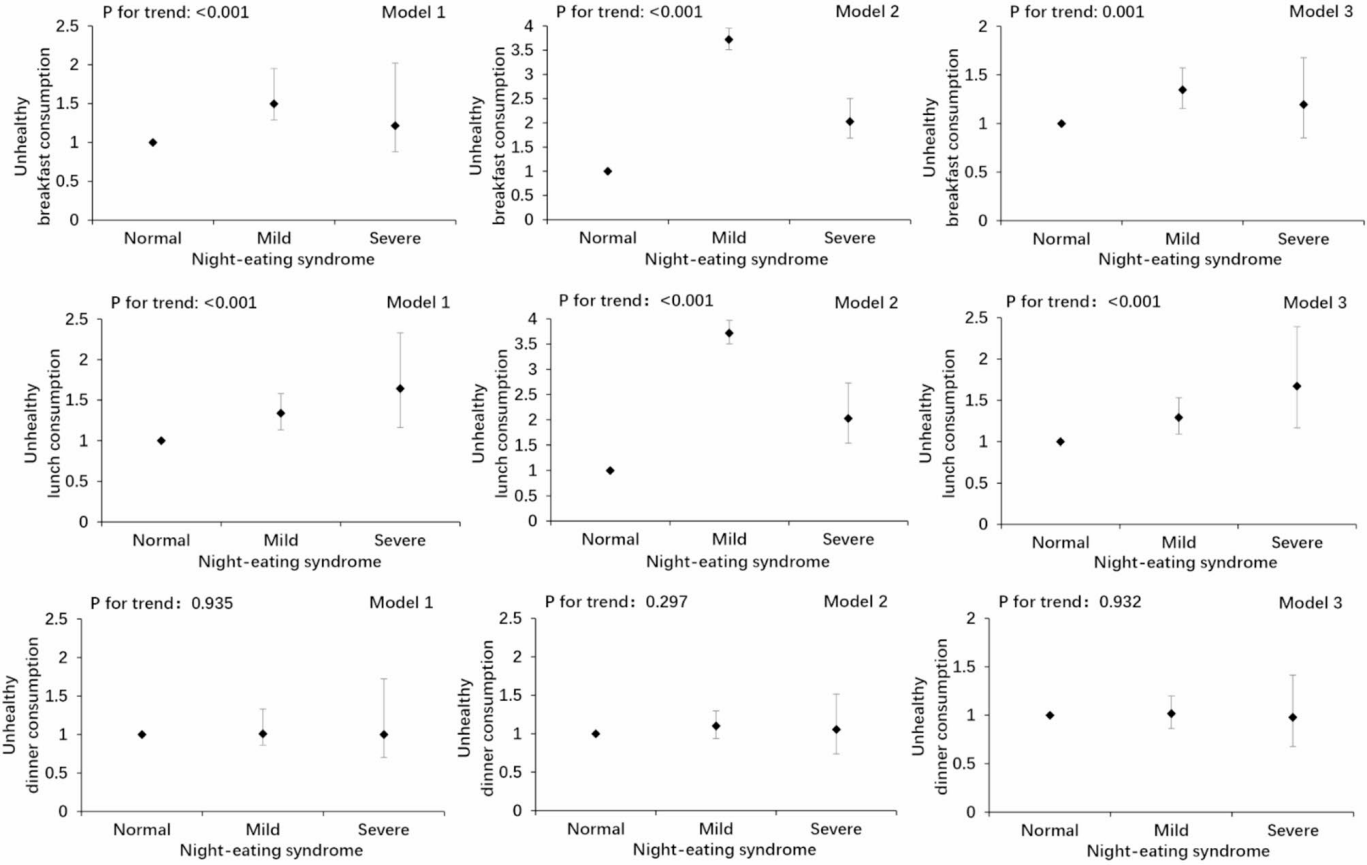
Multivariate logistic regression of meal frequency according to night eating syndrome. Model 1: crude; Model 2: adjusted for sex, age, annual family income (< 20000, 20000–35000, > 35000 yuan), place of residence (town or rural), and parental education (primary and below, junior high, high school, or junior college and above); Model 3: adjusted for Model 2 variables plus sleep duration, sleep quality, and depression index. Adjusted data are expressed as odds ratio (95% confidence intervals).

After adjusting for potential confounders, this study found statistically significant associations between NES levels and several dietary and meal frequency outcomes. Specifically, higher frequency of fruit consumption classified as unhealthy according to the scoring criteria.was associated with NES (mild group: OR = 3.796, 95% CI: 3.264–4.414; severe group: OR = 2.674, 95% CI: 1.899–3.763;P for trend < 0.001). The same was observed for vegetable consumption frequency (mild group: OR = 2.773, 95% CI: 2.386–3.222; severe group: OR = 1.865, 95% CI: 1.345–2.586༛P for trend < 0.001), snack consumption frequency (mild group: OR = 0.593, 95% CI: 0.499–0.705; severe group: OR = 1.240, 95% CI: 0.889–1.729༛P for trend = 0.001), soft drink consumption frequency (mild group: OR = 0.633, 95% CI: 0.542–0.740; severe group: OR = 1.195, 95% CI: 0.867–1.648; P for trend = 0.001), irregular breakfast consumption (mild group: OR = 1.347, 95% CI: 1.155–1.572; severe group: OR = 1.196, 95% CI: 0.852–1.677; P for trend = 0.001), and irregular lunch consumption (mild group: OR = 1.292, 95% CI: 1.090–1.531; severe group: OR = 1.671, 95% CI: 1.166–2.394; P for trend < 0.001) compared with the normal group. However, no significant associations were observed between NES levels and the consumption frequency of other food items or dinner frequency (Figs. 1 and 2).

## Discussion

To date, studies on the effect of NES on food consumption frequency have primarily focused on the association between NES and a single food consumption frequency^7,8^ or the frequency of three meals per day^5,9,10^. To our knowledge, this is the first study to examine the associations between NES and food consumption frequency as well as meal frequency in college students. Our findings indicate that individuals with NES tended to report a higher consumption frequency for high-calorie foods, such as sugary beverages and snacks, and lower consumption frequencies for vegetables and fruits compared with those without NES. Conversely, students without NES were more likely to maintain regular breakfast and lunch patterns than those with NES. These findings are consistent with those from previous investigations. However, in contrast to previous studies, we used a representative sample of Chinese university students to comprehensively examine the association between NES and a range of food consumption frequencies and meal frequencypatterns.

Regarding potential mechanisms underlying the association between NES and food consumption frequency, it is plausible that endogenous circadian rhythms mediate this effect. Hunger exhibits a circadian rhythm, peaking in the evening (around 8 p.m.) and reaching its lowest point in the morning (around 8 a.m). The influence of circadian rhythms on the appetite for specific food groups was is more pronounced in the evening, which is associated with an increased desire for high-energy foods such as sweets, salty snacks, carbohydrates, meat, poultry, and fruits. In contrast, there is less evidence of a circadian influence on the appetite for low-energy foods such as vegetables^17^. Thus, individuals with NES, characterized by nocturnal eating, may experience a heightened evening appetite driven by circadian rhythm, contributing to increased intake of high-energy foods^18^ and reduced appetite during the day, leading to irregular meal patterns^19^.

Changes in appetite-related hormones, such as leptin, may also play a role. Sleep suppresses appetite by prompting the release of leptin, which is produced by adipocytes^20,21^. This suppression persists during the morning wake state. In contrast, NES is characterized by nighttime and nocturnal hyperphagia, morning purging, and insomnia, accompanied by a reduction in leptin levels during nocturnal sleep periods. This may result in increased hunger and subsequent food intake^22^, as supported by a cross-sectional study showing a significant positive association between NES and leptin levels^23^.

Beyond the biological mechanisms, behavioral and lifestyle factors specific to university students may further amplify the association between NES and food consumption frequency. For instance, academic pressure can cause worry, which may lead to overthinking or engaging in entertainment activities, such as using phones or tablets, that can delay bedtime^24,25^ and mealtimes^26,27^, increase cognitive load^28^, raise energy expenditure^29^, and ultimately stimulate appetite for high-energy foods^30^.

Notably, this study identified a significant association between severe NES and reduced frequency of breakfast and lunch consumption. The schedules of Chinese university students may be a contributing factor to the difficulties experienced by nocturnal eaters in consuming breakfast and lunch. Breakfast is typically served between 7 a.m. and 9 a.m. The schedules of the first and second classes (at 8 a.m. and 10 a.m., respectively) influence students’ eating habits, particularly for nocturnal eaters, who may have no choice but to eat between classes. Furthermore, the proximity of breakfast to lunchtime makes it challenging for nocturnal eaters to reach sufficient hunger to eat lunch. In a study of Chinese university students (both male and female), approximately 40% consumed breakfast at least 30 min after awakening, whereas approximately 20% did not consume breakfast at the optimal time^31,32^.

This study has some limitations that should be considered when interpreting the findings. First, the cross-sectional design precludes the determination of causal relationships between NES, food consumption frequency, and meal frequency. Second, the self-reported nature of meal frequency data, collected based on participants’ recall of generic meal categories (breakfast, lunch, and dinner) without reference to specific timeframes, may introduce recall bias and lead to misclassification of meal patterns. Future research should employ more precise dietary assessment tools, such as 24-h dietary recalls, food frequency questionnaires, or food diaries, to evaluate the quantity and quality of food consumption more accurately. Third, although the depression scale used in this study includes items that conceptually overlap with stress and anxiety symptoms, these individual questions cannot fully assess a person’s stress or anxiety levels. Therefore, future studies should incorporate validated questionnaires assessing anxiety and stress to clarify whether these factors, as potential confounders, affect the association between NES and eating behaviors.

In conclusion, the results suggest a strong association between NES and food consumption frequency and meal regularity. Individuals with severe NES may exhibit irregular meal patterns and increased consumption of high-energy foods. These findings emphasize the importance of interventions targeting NES, which may help students modify their food choices and establish consistent habits of consuming three balanced meals daily. Future research should employ longitudinal, prospective, or randomized controlled designs to validate and extend these findings.

## Data Availability

The datasets used and/or analyzed during the current study are available from the corresponding author on reasonable request.
